# Gestational Diabetes Mellitus Is Associated with Altered Neutrophil Activity

**DOI:** 10.3389/fimmu.2017.00702

**Published:** 2017-06-14

**Authors:** Maria Stoikou, Franco Grimolizzi, Stavros Giaglis, Günther Schäfer, Shane Vontelin van Breda, Irene Mathilde Hoesli, Olav Lapaire, Evelyn A. Huhn, Paul Hasler, Simona W. Rossi, Sinuhe Hahn

**Affiliations:** ^1^Department of Biomedicine, University of Basel, University Hospital of Basel, Basel, Switzerland; ^2^Department Clinical Sciences, Polytechnic University Marche, Ancona, Italy; ^3^Department of Rheumatology, Kantonsspital Aarau, Aarau, Switzerland; ^4^University Women’s Hospital, University Hospital of Basel, Basel, Switzerland

**Keywords:** neutrophil extracellular traps, gestational diabetes mellitus, pregnancy, IRS1, TNFa, citH3

## Abstract

Gestational diabetes mellitus (GDM) is a unique form of glucose intolerance, in that it is transient and solely occurs in pregnancy. Pregnancies with GDM are at high risk of developing preeclampsia (PE), a leading cause of fetal and maternal morbidity or mortality. Since PE is associated with excessive activation of circulatory neutrophils and occurrence of neutrophil extracellular traps (NETs) in affected placentae, we examined these features in cases with GDM, as this could be a feature linking the two conditions. Our data indicate that neutrophil activity is indeed altered in GDM, exhibiting pronounced activation and spontaneous generation of NETs by isolated neutrophils in *in vitro* culture. In this manner, GDM may similarly affect neutrophil behavior and NET formation as witnessed in other forms of diabetes, with the addition of the physiological changes mediated by pregnancy. Since circulatory TNF-α levels are elevated in cases with GDM, a feature also observed in this study, we examined whether this pro-inflammatory cytokine contributed to neutrophil activation. By using infliximab, a clinically utilized TNF-α antagonist, we observed that the pro-NETotic effect of GDM sera was significantly reduced. We also detected pronounced neutrophil infiltrates in placentae from GDM cases. The occurrence of NETs in these tissues is suggested by the extracellular co-localization of citrullinated histones and myeloperoxidase. In addition, elevated neutrophil elastase (NE) mRNA and active enzymatic protein were also detected in such placentae. This latter finding could be important in the context of previous studies in cancer or diabetes model systems, which indicated that NE liberated from infiltrating neutrophils enters surrounding cells, altering cell signaling by the degradation of IRS1. These findings could potentiate the underlying inflammatory response process in GDM and possibly open an avenue for the therapeutic interventions in gestational hyperglycemia.

## Introduction

Gestational diabetes mellitus (GDM) is a unique form of glucose intolerance exclusive to pregnancy and that by nature it is transient (1, 2). Since GDM may share some similarities with Type 2 diabetes mellitus (T2DM), such as insulin resistance or features of the metabolic syndrome, it has been suggested that GDM may be considered a pre-diabetic state or a momentary unmasking of a T2DM-like condition (2). Additionally, the autoimmune component evident in Type 1 diabetes mellitus (T1DM) is not evident in GDM (1, 2). Fueled by the pandemic in obesity and associated metabolic syndrome, GDM is rapidly becoming a global health-care concern, particularly as it may significantly increase the incidence in T2DM due to the contribution of two parties, mother and child (3). In order to be able to undertake effective countermeasures to avert or reduce this scenario, a more precise knowledge of the underlying etiology is required to facilitate the development of efficacious therapeutic strategies (4).

Although therapies exist to manage GDM (4–7), affected pregnancies are associated with considerable maternal and fetal morbidity, being at particular risk for preeclampsia (PE). PE is a severe hypertensive disorder of the latter part of pregnancy (8–10) characterized by wide-spread endothelial dysfunction mediated largely by deregulation of placentally produced angiogenic factors (11). PE also involves a pronounced inflammatory component, largely evident by excessive neutrophil activation (12, 13). Neutrophils, also termed polymorphonuclear neutrophil granulocytes (PMN), are currently experiencing a renaissance of scientific interest in a diverse array of human pathologies, largely due to their ability to form neutrophil extracellular traps (NETs) (14–16).

Originally described as a novel means to ensnare and kill invasive pathogens, NETs are formed by the extrusion of nuclear DNA coated with toxic granular components into the extracellular milieu (17), *via* a novel form of cell death termed NETosis.

Prompted by our previous work on the occurrence of elevated levels of cell-free DNA in the circulation of pregnant women with PE (18, 19), we examined whether this disorder is associated with aberrant NETosis and whether such errant neutrophil activity could be the source of this increase in extracellular circulatory DNA. In these investigations, we detected the occurrence of large numbers of NETs in affected placentae (20). This was the first report of aberrant NETosis in a human pathology not related to infection. As NETs were detected directly in the intervillous space, this could lead to occlusion, or infarction, thereby contributing to placental insufficiency proposed to be involved in the underlying etiology of PE (21). Interestingly, NETs were also recently detected in cases of PE associated with systemic lupus erythematosus (SLE) (22), suggesting that these structures may indeed contribute to the development of this enigmatic disorder.

Subsequent studies have indicated that aberrant NET formation may contribute to the etiology of a number of inflammatory conditions including rheumatoid arthritis (RA), SLE, small-vessel vasculitis, coagulopathies, and possibly assist with the metastasis and growth of tumor cells (16, 20, 23–26).

NETosis also appears to be altered in cases with diabetes [for a recent review, refer to Ref. (15)]. Such an instance was first noted when investigating infections with *Burkholderia pseudomallei*, which appear to be more prevalent in diabetic patients due to a defect in NETosis (27). Joshi et al. noted that while culture (24 h) of isolated neutrophils under high glucose (HG) conditions (30 mM) led to increased NET formation. This was reduced upon subsequent treatment with either LPS, PMA, or TNF-α (28), a feature also recently observed by Carestia and colleagues in an examination of neutrophils isolated from cases with T2DM (29). This is in contrast to the report by Menegazzo and colleagues, who observed that treatment of freshly isolated neutrophils with HG (25 mM) led to enhanced spontaneous NET formation in culture, which was further augmented upon treatment with PMA (30). These authors also noted that the concentrations of NETs or neutrophil-derived products, such as circulating nucleosomes, dsDNA, or neutrophil elastase (NE), were elevated in the plasma of T2DM patients when compared to controls (30). The latter approach was exploited by Wang and colleagues in a study to determine whether NETosis was altered in cases with T1DM (31) and in recent examinations of NETosis in cases with T2DM undergoing therapy (29, 32). While the use of such assays will no doubt remain contentious, and while it is indisputable that such markers are not exclusively of NETotic origin, a number of other studies have suggested they can serve as surrogates to monitor NETs activity under a variety of inflammatory conditions, including RA or PE (24, 33).

Overt NETosis in diabetes may, however, have other consequences, such as delayed or impaired wound healing, as suggested by two recent reports examining the influence of NET formation on skin wound repair or diabetic foot ulcers (34, 35).

A consensus of these diverse reports is that hyperglycemic conditions promote a pro-NETotic response, which can be augmented by secondary stimuli (15).

The contribution of neutrophils to the etiology of disorders and their development is not restricted to NET formation, but can involve the uptake of toxic granular enzymes, such as NE by surrounding tissues (36, 37). In the first such report, it was noted that the presence of tumor-infiltrating neutrophils can promote tumor cell growth by a mechanism involving NE (36). In this instance, it was determined that exogenous NE released by degranulation was absorbed by surrounding cancer cells where its enzymatic activity led to the degradation of a key regulatory molecule, IRS1. This led to uncoupling of the PI3K signaling pathway, thereby promoting uncontrolled cell growth (36).

The second report regarding such an interaction of exogenous NE with surrounding tissues was made by Talukdar and colleagues using a murine model of high-fat diet-induced diabetes (T2DM) (37). In this study, it was demonstrated that neutrophils contribute to the development of insulin resistance in hepatocytes by the release of NE, which alters AMPK signaling *via* IRS1 degradation (37).

In a recent study, we determined that pregnancy is characterized by pro-NETotic activation, mediated largely by elevated circulatory concentrations of G-CSF and modulated by estrogen and progesterone (38), thereby illustrating that the underlying host physiology plays a significant role in modulating the immune response (39). As NETosis is altered in cases with T1DM or T2DM, we now examined this aspect in pregnancies affected by GDM, especially as such pregnancies are at increased risk of developing PE (9, 10). Since aberrant NETosis is implicated in the underlying etiology leading to the development of PE, evidence of a similar alteration in GDM may provide insight into a mechanism linking these two pathologies (20, 22, 33).

## Materials and Methods

### Human Subjects

This study was carried out with the approval of the Ethical Committee Nordwest und Zentralschweiz, Basel, Switzerland (EKBB 195/13 or EKNZ PB 2016-00611), with written informed consent from all subjects in accordance with the Declaration of Helsinki.

Pregnant women (*n* = 19: *n* = 10 healthy pregnant and *n* = 9 GDM pregnancy) were recruited at the time of their routine examination for glucose intolerance between the 24th and 28th weeks of pregnancy (median gestational age: 25 weeks and 6 days; median age: 34.5 years). Healthy non-pregnant controls matched for age (*n* = 10) were recruited at the Blood Bank of the Swiss Red Cross, Basel. Human placentae were obtained from pregnant women who delivered healthy, singleton infants at term (>37 weeks’ of gestation) undergoing elective cesarean section.

Inclusion criteria for the pregnant subjects included healthy singleton pregnancies. Exclusion criteria included maternal diseases like hypertension, diabetes mellitus and chronic disease, known infection like hepatitis or human immunodeficiency virus, maternal history of hypertensive diseases in previous pregnancy, and fetal genetic, chromosomal or intervention-requiring morphologic abnormalities. Inclusion criteria for non-pregnant controls were fair general condition, female sex, age ≥25 and ≤45 years and for blood donors fulfilling national criteria for blood donation. Exclusion criteria were current or previous systemic autoimmune disease, asthma, convalescence after major illness, surgery, current medication with corticosteroids, immunosuppressive agents, and malignant neoplasia or chemotherapy within 5 years before recruitment for the study.

Blood samples from patients affected by TD1 and TD2 were collected during routine visits and directly processed for analysis.

### Sample Collection and Processing

Whole blood was collected into EDTA- and silicone-coated tubes (Sarstedt) and analyzed by Hemavet 950FS (Drew Scientific) for complete blood cell counts. Plasma and serum were collected and processed as described previously (24, 33, 38). Samples were studied immediately or stored at −80°C until analyzed.

Five placentae samples for each group were collected and dissected from the middle cross section of villus tree within 15 min of the delivery. To compensate for intra-placental variability, we collected 3 independent samples per placenta giving a total of 15 GDM and 15 control samples. Tissue was bluntly dissected to remove visible connective tissue, blotted dry on filter paper, snap frozen in liquid nitrogen, and stored at −80°C. Small pieces of tissues were embedded in optimal cutting temperature compound (Tissue-Tek; Sakura Finetek USA) and kept at −80°C until cryosection.

### Oral Glucose Tolerance Test (OGTT)

Pregnant women between the 24th and 28th weeks of gestation, after 10–16 h of fasting, gave blood, and the fasting glucose levels were tested. They ingested 300 ml of Accu-Chek Dextrose O.G.-T. (Roche), which contains 75 g of glucose, and gave blood after 60 and 120 min, according to manufacturer’s instructions. Glucose was measured in the certified laboratory of the University Hospital Basel.

### Human Neutrophil Isolation

Neutrophils were isolated by Dextran-Ficoll density centrifugation (20, 40). Cell viability was assessed by trypan blue dye exclusion in a hemocytometer and was routinely 96–98% with a purity of over 95%. Neutrophils were directly seeded in 24-well or 96-well plates and allowed to settle for 15 min at 37°C under 5% CO_2_ prior to further experimentation.

### BeWo Cells and Coculture Conditions

BeWo cells (ATCC CCL-98) were grown at 37°C under a humidified 5% CO_2_/95% air atmosphere in F-12K Medium containing 10% fetal bovine serum (FBS) and 1% penicillin/streptomycin. Experiments were performed under serum-starved conditions using cells at 80% confluence between passages 30 and 40. BeWo cells were grown under low glucose (7 mM) or HG (25 mM) conditions. Supernatants were harvested, submitted to centrifugation, aliquoted, and frozen at −80°C until use. The coculture experiments were performed in 6-well cell culture plates with 12 mm coverslips (Corning^®^ BioCoatTM Coverslips). When indicated, neutrophils were directly added to the BeWo cells at a density of 0.5 × 10^6^ neutrophil/ml for 3 h. In neutralization studies, 5 µg/ml of chimeric anti-TNF-α IgG1 antibody infliximab (Remicade, MSD) was given simultaneously to neutrophil addition.

### Fluorimetric Quantification and Fluorescence Microscopy

Neutrophil extracellular traps were quantified by SytoxGreen fluorimetry (20, 40). Neutrophils (2.5 × 10^4^) freshly isolated were cultured in the presence of 0.2 µM SytoxGreen (Invitrogen, Life Technologies) in a 96-well dark microtiter plate at 37°C under 5% CO_2_ and left untreated or stimulated with the indicated agents over 3 h. PMA was used as the positive control. Fluorescence (excitation 485 nm, emission 535 nm) was measured in a Biotek Synergy H1 Hybrid Reader (Biotek) and results given as mean DNA fluorescence intensity. Photomicrographs in bright field and green fluorescence spectra were assessed with an Olympus IX50 inverted fluorescence microscope coupled to an Olympus XM10 monochromatic CCD camera and analyzed with the Olympus CellSens Dimension software (Olympus).

### Immunohistochemistry and Morphometric Analysis

Neutrophil extracellular traps were quantified by IHC staining of 2.5 × 10^4^ neutrophils per well in a 96-well plate, mouse anti-human MPO antibody (1:750, ab25989, Abcam) and rabbit anti-human citH3 antibody (1:150, ab5103, Abcam), or the respective isotype controls, followed by incubation with goat anti-mouse IgG AF555 (1:1,000, A28180, Invitrogen Life Technologies) and goat anti-rabbit IgG AF488 (1:1,000, A11034, Invitrogen Life Technologies) (24, 38). DNA was counterstained with 4′,6-diamidino-2-phenylindole (DAPI, D9542, Sigma-Aldrich). NETs were visualized by using an Olympus IX81 motorized epifluorescence microscope (Olympus) in conjunction with an Olympus XM10 monochromatic CCD camera (Olympus) and analyzed with the Olympus CellSens Dimension software (Olympus). A minimum of 20 fields at 10× magnification (at least 500 to 1,000 neutrophils) per sample were evaluated for MPO/citH3 and DNA co-staining through ImageJ analysis software (NIH); nuclear phenotypes and NETs were determined, counted, and expressed as percentage of the total area of cells in the fields. For staining and quantification of NE, 1 × 10^5^ neutrophils were seeded on poly-l-lysine-coated glass coverslips (BD Biosciences) in 24-well tissue-culture plates, fixed with 4% paraformaldehyde, and blocked overnight (HBSS with 10% FBS, 0.1% Tween20, and 2 mM EDTA) at 4°C. Samples were stained with rabbit anti-NE (1:200, ab26154, Abcam) following incubation with goat anti-rabbit IgG AF488 (1:1,000, Invitrogen Life Technologies). DNA was stained with DAPI (1:10,000, Sigma-Aldrich). Coverslips were visualized using an Olympus BX61 Diana fluorescent microscope.

For double immunohistochemistry of the human placentas, 8-µm thick placental sections were fixed in 4% paraformaldehyde, permeabilized with 0.2% Triton X-100, and blocked in 3% BSA overnight. Primary antibodies used were: rabbit anti-citH3 (1:200, Abcam), rabbit anti-MPO (1:200, Abcam), mouse anti-NE (1:200, HM2174, Hycult Biotech), and rabbit anti-IRS1 (1:200, sc-559, Santa Cruz). Sections were incubated in a light-protected humidified chamber with Alexa 488-conjugated goat anti-rabbit (1:1,000, Life Technologies) and Alexa 647-conjugated goat anti-mouse (1:1,000, A21237, Life Technologies) secondary antibodies. Sections were stained with DAPI (1:10,000) and mounted in Mowiol (Sigma-Aldrich). In addition, a further consecutive section was stained using H&E for comparison against immunostained tissues. The slides were observed under an Olympus BX61 Diana fluorescent microscope and photographed.

The cells, following coculture experiments, were fixed in 2% paraformaldehyde and blocked with 3% BSA. Primary antibodies used were: rabbit anti-citH3 (1:150, Abcam) and mouse anti-NE (1:150, Hycult Biotech), or the respective isotype controls, followed by incubation with goat anti-mouse IgG AF555 (1:1,000, A21424) and goat anti-rabbit IgG AF488 (1:1,000, A11034, Invitrogen Life Technologies). DNA was counterstained with DAPI (1:10,000).

### Cell-Free Nucleosome, NE, MPO, A1AT, and TNF-α ELISA

Histone/DNA complexes in sera and plasma were measured using the Human Cell Death Detection ELISA^PLUS^ (Roche Diagnostics) (24, 38). The concentrations of NE in sera and plasma were measured by sandwich ELISA, utilizing the Elastase/a1-PI Complex ELISA Kit (Calbiochem) (24, 38). For the detection of MPO in sera and plasma, the Hycult Biotech ELISA Kit was used (24, 38). For the detection of A1AT in sera and plasma, the AssayPro ELISA Kit was used. TNF-α concentrations in sera and plasma were assessed by sandwich ELISA with Human TNF-α DuoSet ELISA Kit (R&D Systems).

### Stimulation and Neutralization Studies

For *in vitro* incubation studies, 2.5 × 10^4^ neutrophils from healthy women were treated with 6% plasma derived from non-pregnant controls, pregnant donors, and donors with GDM during second and third trimesters of gestation (24, 38). All experiments were carried out over 3 h in four to six replicates.

For similar *in vitro* incubation studies, 2.5 × 10^4^ neutrophils from healthy women were treated with supernatants from BeWo cells after exposure to normal or high glucose for 12 h.

To neutralize TNF-α, pooled plasma from the study groups of interest or BeWo supernatant was pretreated for 30 min with Infliximab (5 µg/ml).

For the two-step stimulation *in vitro* experiments, neutrophils from healthy controls were pretreated or not with TNF-α (50 ng/ml) for 30 min and then exposed to HG (25 mM) for 150 min, for a total time of 3 h.

### Protein Isolation and Western Blotting

Total cellular protein was extracted using Pierce RIPA Buffer (Thermo Scientific) supplemented with PhosSTOP Protease Inhibitor Cocktail Tablets (Roche Life Science). For the placenta, frozen tissues were thawed for several minutes in pre-chilled RIPA buffer and homogenized with a Polytron (Kinematica). Western blotting was performed by using AnykD Mini-PROTEAN TGX Gels (Biorad, Hercules, CA, USA) and nylon/nitrocellulose membranes (Biorad). Primary and secondary antibodies used were: rabbit anti-IRS1 (1:500, sc-559, Santa Cruz Biotechnology), rabbit anti-GLUT4 (1:1,000, 07-1404, Millipore), mouse anti-β-Actin (1:5,000, Sigma-Aldrich), goat anti-Mouse (sc-2005, Santa Cruz Biotechnology), and/or anti-Rabbit (sc-2004, Santa Cruz Biotechnology), human anti-HRP (1:2,000, Santa Cruz Biotechnology). HRP activity was detected by using SuperSignal West Pico Chemiluminescent Substrate (Thermo Scientific). Densitometric analysis and protein quantification of the western blots was performed by using Image Lab Software (version 5.2.1). Blots were stripped briefly in 0.2 M sodium hydroxide and reprobed for mouse anti-β-Actin (Sigma-Aldrich) as a loading control.

### RNA Isolation and Quantitative Real-time PCR

Total RNA was isolated from 3 × 10^6^ neutrophils by using the RNeasy Mini Kit (Qiagen). TaqMan real-time quantitative RT-PCR was performed utilizing the Applied Biosystems StepOne Plus cycler (Applied Biosystems) and TaqMan Gene Expression Assay primer and probe sets (Applied Biosystems) for ELANE (HS00236952_m1), TNF (HS01113624_g1), and RPLP0 (HS99999902_m1). Data were normalized to the housekeeping gene B2M (HS99999907_m1), after a selection procedure from six different endogenous reference genes, as suggested in the MIQE guidelines. Relative values were calculated with 2^−DDCt^ analysis.

### Radiometric Glucose Uptake Assay

BeWo cells were preincubated with 80 nM of human NE (SE563, Elastin products company) in glucose-free KrebsRinger HEPES buffer (KRH) [25 mM Hepes-NaOH (pH 7.4), 120 mM NaCl, 5 mM KCl, 1.3 mM CaCl_2_, 1.3 mM KH_2_PO_4_] for 3 h. Then, cells were rinsed with KRH and stimulated in the same buffer containing 100 nM of insulin (Actrapid HM) for 1 h. Following stimulation, the cells were washed and in order to measure the glucose uptake, incubated in KRH buffer supplemented with 1 μCi/ml of 2-[1,2-3H(N)]-deoxy-d-glucose (Perkin Elmer) for 10 min. Resulting cell lysate was mixed with scintillation fluid (Filter-count, Perkin Elmer), and radioactivity was measured in a liquid scintillation analyzer (Packard 1900 TR). Glucose uptake was expressed in triplicate, and the mean SD of the data was calculated.

### Statistical Analysis

All data are presented as mean ± SEM. Descriptive statistics for continuous parameters consisted of median and range, and categorical variables were expressed as percentages. Comparisons between patients and healthy controls were carried out by the Mann–Whitney *U* test with a Welch post-test correction. Statistical significance in multiple comparisons was by one-way analysis of variance (ANOVA) with a Dunn’s post-test correction. *P* values <0.05 were considered statistically significant. Data were processed in GraphPad Prism version 6.0 for MacOSX (GraphPad Software Inc.).^1^ Andreas Schoetzau provided professional statistical assistance.^2^

## Results

### GDM Is Associated with Excessive NETosis and NE Liberation

**Figure 1:**
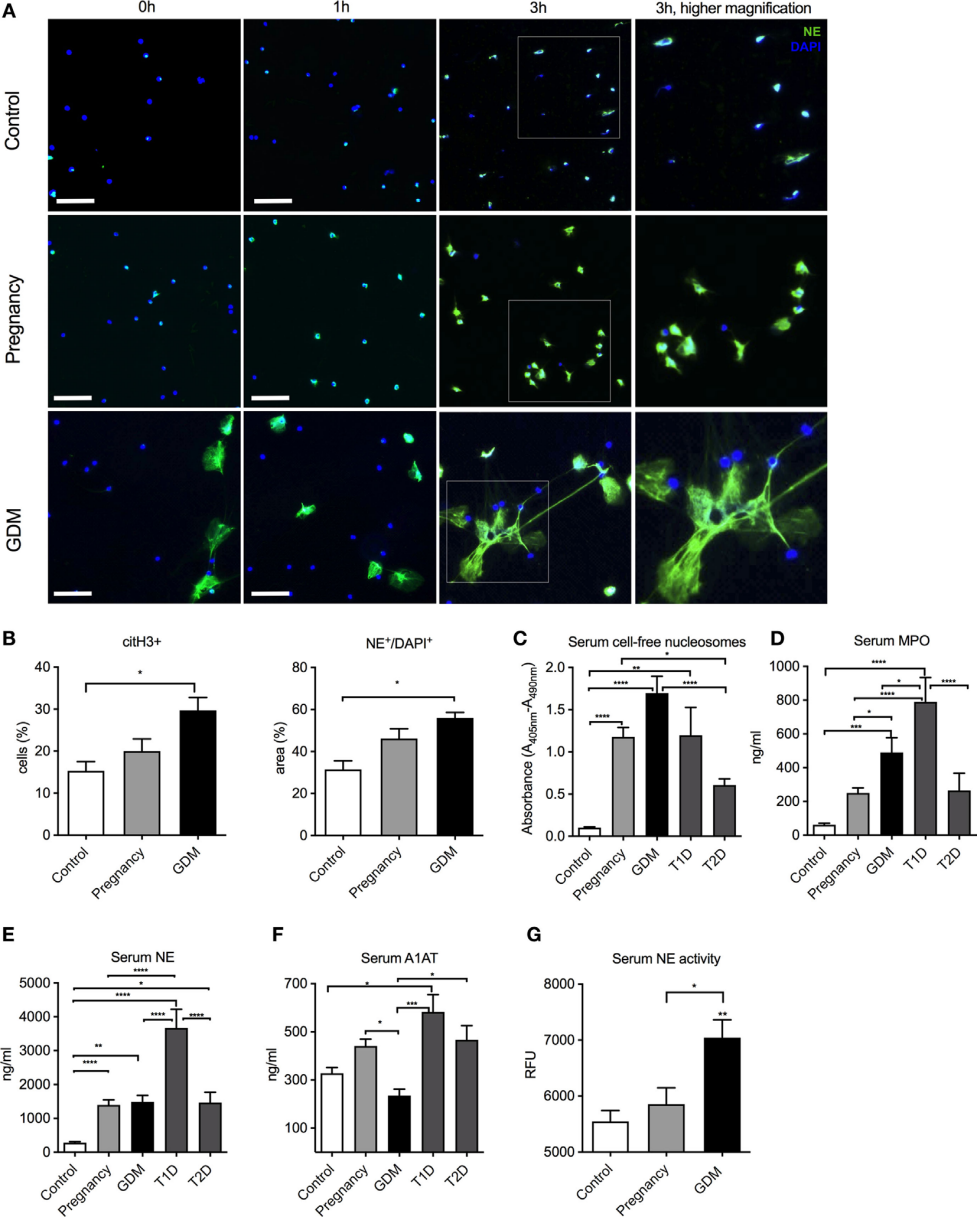
Pronounced neutrophil extracellular trap (NET) formation in pregnancies is affected by gestational diabetes mellitus (GDM). **(A)** *In vitro* spontaneous NET formation assay performed with neutrophils isolated from non-pregnant (controls), healthy pregnant (pregnancy), and GDM patients during a 3-h time course. NETs are detected by immunofluorescence staining for neutrophil elastase (NE) (green) and DNA-DAPI (blue). Scale bars: 50 µm. **(B)** Morphometric analysis of extracellular NETs with citH3 staining (% of positive cells) and NE^+^/DAPI^+^ staining (% positive area) from controls, pregnancy, and GDM neutrophils. **(C–E)** Measurement of cell-free nucleosomes of control, IInd, and IIId trimester pooled pregnancy samples, IInd and IIId trimester pooled GDM samples, type 1 diabetes mellitus (T1DM), and type 2 diabetes mellitus (T2DM) serum samples **(C)**, myeloperoxidase **(D)** and NE **(E)**. **(F)** Quantitation of serum A1AT levels in controls, pregnancy, GDM, T1DM, or T2DM. **(G)** Serum NE enzymatic activity in controls, pregnancy, and GDM. **(B–G)** Data are presented as mean ± SEM (*P < 0.05, **P < 0.01, ***P < 0.001, ****P < 0.0001) (one-way ANOVA followed by Tukey’s multiple comparison post-test). All experiments were performed at least six times with consistent results.

In an exploratory examination of the NETotic activity of freshly isolated neutrophils *in vitro*, we observed that NET formation was significantly elevated in a case with GDM when compared to a matching second trimester sample (Figure 1A). This feature was confirmed in a larger series of GDM cases (*n* = 9) and matching healthy control pregnancies (*n* = 10) and non-pregnant donors (*n* = 10) (Table S1 in Supplementary Material; Figure 1B). In these analyzes, it was noted that GDM samples contained more neutrophils activated to undergo NETosis as apparent from the presence of citrullinated histone H3 (citH3 +) moieties (Figure 1B), a key feature in the NETotic signaling cascade. This was mirrored in a greater tendency to undergo NETosis *in vitro*, as indicated by the quantity of extracellular NE/DNA (total area) (Figure 1B, lower panel). Furthermore, this NETosis increased from the time of diagnosis (24–28 weeks of gestation) throughout the second and third trimesters of gestation as measured with cell-free nucleosomes in the supernatant of freshly isolated neutrophils (Figure S1 in Supplementary Material).

Since numerous reports indicate that NETosis is elevated in cases with T1DM or T2DM (15), we compared such activity to that in cases with GDM, using serum cell-free DNA (nucleosomes) as an indicator for NETs release. Although this parameter does not specifically detect NET-derived products in human or murine body fluids, it has been widely used as a surrogate marker for predisposition to NETosis in RA, PE, coagulation disorders, cancer patients, or T1DM and T2DM (24, 29, 31, 33, 41–44). Neutrophils from cases with GDM exhibited the greatest tendency to undergo NETosis, being significantly higher than cases with either T1DM or T2DM (Figure 1C). These results also reaffirmed that pregnancy *per se* is a pro-NETotic state (Figure 1C) (33). We, next, examined whether other indicators of aspects of neutrophil activity were altered, such as the release of granular enzymes MPO or NE. These were the highest in cases with T1DM (Figures 1D,E).

We were, however, puzzled by the observation that NE release by degranulation was so low in cases with GDM, when compared to normal pregnant controls (Figure 1E). As the EIA assay employed in this analysis detects a complex of NE with its native inhibitor A1AT, we examined the circulating concentrations of the latter, which indicated that these were significantly lower in GDM cases (Figure 1F). As this could lead to an incorrect measurement of NE levels, we accordingly assessed for NE enzymatic activity, which clearly indicated that elastase enzymatic activity was significantly higher in GDM cases than normal pregnant controls (Figure 1G).

### TNF-α Promotes Pro-NETotic Activation in GDM

**Figure 2:**
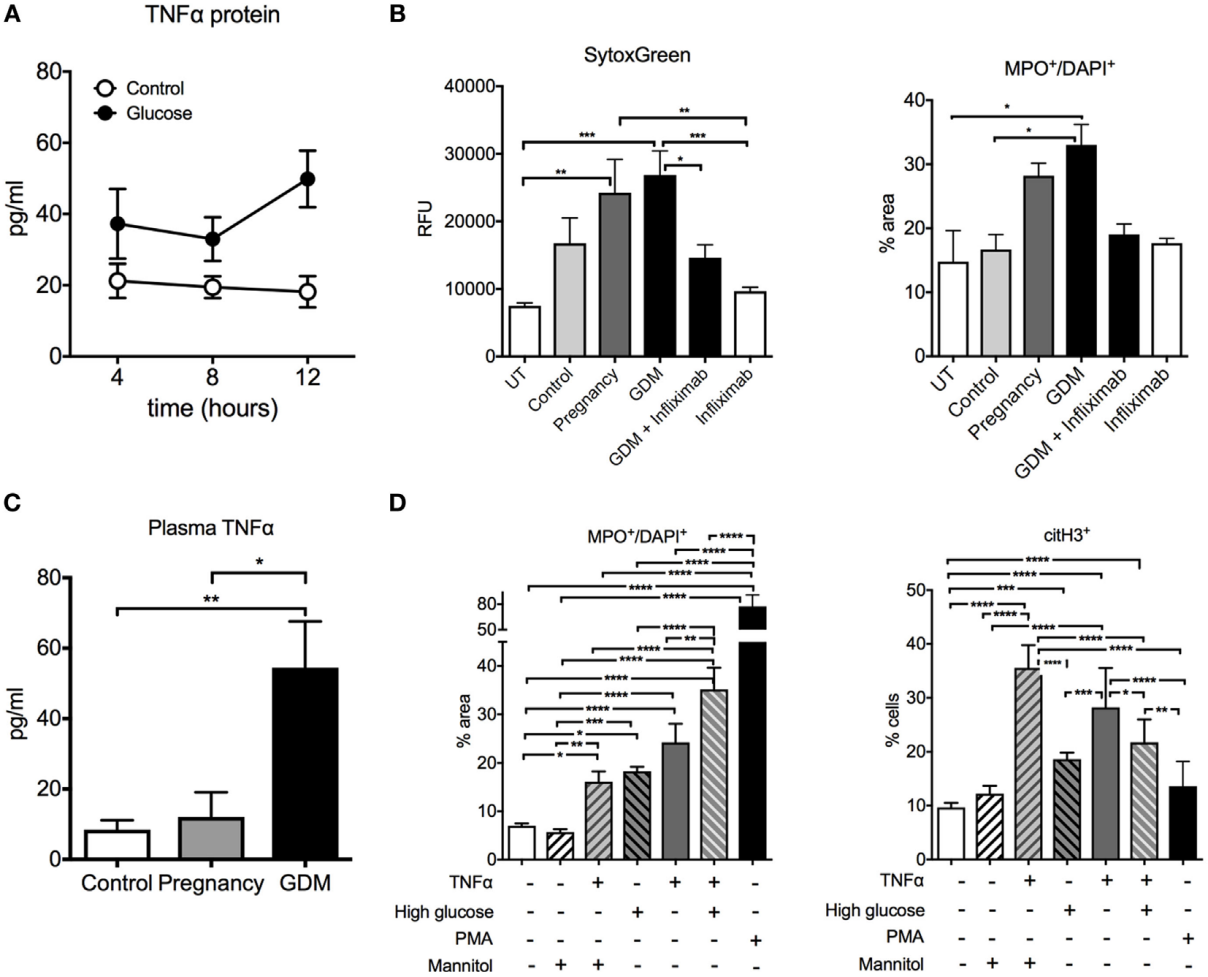
Glucose and TNF-α induce neutrophil activation and neutrophil extracellular traps (NETs) formation in gestational diabetes mellitus (GDM). **(A)** TNF-α concentration in the supernatants from BeWo cells treated with/without 25 mM of d-glucose. **(B)** NET morphometry over a 3-h time course with SytoxGreen DNA-binding dye (left panel) and MPO^+^/DAPI^+^ (right panel) after *in vitro* incubation of control neutrophils with control, pregnancy, and GDM plasma with and without pretreatment with 5 µg/ml infliximab. **(C)** TNF-α concentration of controls, pregnancy, and GDM plasma. **(D)** Morphometric quantitation of NETs performed with MPO^+^/DAPI^+^ and citH3 of neutrophils from controls after exposure to 25 nM glucose, 25 nM Mannitol, 50 ng/ml TNF-α, and PMA 50 ng for 3 h. **(A–D)** Data are presented as mean ± SEM (*P < 0.05, **P < 0.01, ***P < 0.001, ****P < 0.0001) (one-way ANOVA followed by Tukey’s multiple comparison post-test). All experiments were performed at least four times with consistent results.

Granted that NETosis and NE release was significantly elevated in GDM, our next task was to gain insight into the factors promoting this change in neutrophil activity. A number of reports have indicated that circulatory levels are elevated in cases with GDM and that the potent pro-inflammatory cytokine TNF-α may assist in promoting a pro-diabetic state (45, 46). Our results indicated that circulatory TNF-α levels were indeed elevated in cases with GDM when compared to matching controls (Figure 2C). Since TNF-α is a potent factor for neutrophil activation and NETosis (47), we examined the effect of plasma from GDM cases and normal healthy control pregnancies. This analysis indicated that plasma from GDM cases promoted a greater degree of NETosis on isolated control neutrophils than comparable plasma from healthy pregnant women (Figure 2B). To confirm that TNF-α played a key role in mediating this pro-NETotic effect, we used infliximab, a specific TNF-α antagonist. Such treatment significantly diminished the pro-NETotic effect of GDM plasma on isolated normal neutrophils (Figure 2B).

It has been suggested that the placenta contributes to the pool of elevated TNF-α, which would explain in part the transient pregnancy associated nature of GDM, which usually resolves shortly after delivery (48, 49).

We examined this issue in a trophoblast cell line, BeWo, where we observed that exposure of these cells to hyperglycemic conditions (25 mM glucose) increased the production of TNF-α (Figure 2A). Hence, these data support a placental contribution to the elevated TNF-α levels in GDM.

Furthermore, TNF-α exerted an additive effect on NETosis induced by hyperglycemia *in vitro* in neutrophils isolated from normal healthy donors (Figure 2D). These findings underscore the potential role of this pro-inflammatory cytokine in neutrophil activation in GDM.

### Glucose Challenge Alters the NETotic Response in GDM

Since an interaction between hyperglycemia and TNF-α on the induction of NETosis was observed *in vitro* (Figure 2D), we examined whether this phenomenon was also evident *in vivo* by analyzing samples taken during the OGTT. This test is routinely used to detect GDM in pregnant women, due to their altered response to a HG challenge, as is evident in our study cohort (Figure S2A in Supplementary Material)(50).

**Figure 3:**
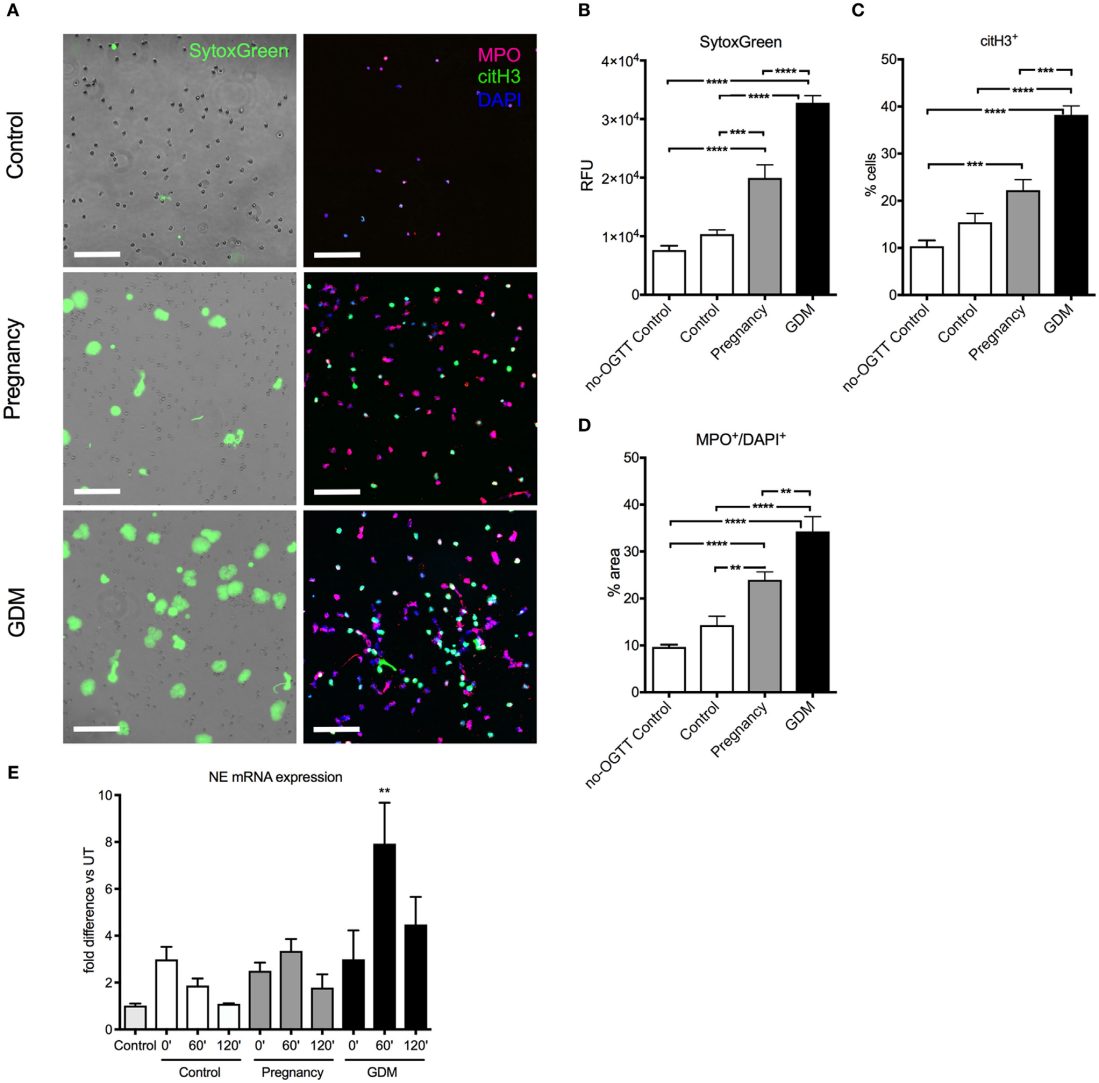
Hyperglycemia induced during oral glucose tolerance test (OGTT) triggers spontaneous neutrophil extracellular trap (NET) formation. **(A)** Spontaneous NET formation in 3-h culture of neutrophils isolated from controls, pregnancy, and gestational diabetes mellitus (GDM) at the 60 min time point of blood collection during OGTT, stained with SytoxGreen (left panel) and immunofluorescence for MPO (red), citH3 (green), and DAPI (blue). Scale bars: 100 µm. **(B)** Fluorimetric analysis with SytoxGreen of NET formation, **(C)** morphometric analysis pro-NETotic primed citH3^+^ and **(D)** NETotic MPO^+^/DAPI^+^ neutrophils isolated during OGTT (60 min). **(E)** mRNA expression of neutrophil elastase (NE) from neutrophils isolated during the OGTT (at 0, 60, and 120 min). Data are presented as mean ± SEM (*P < 0.05, **P < 0.01, ***P < 0.001, ****P < 0.0001) (one-way ANOVA followed by Tukey’s multiple comparison post-test). All experiments were performed at least four times in triplicates with consistent results.

In this instance, clear differences in the NETotic capacity of isolated neutrophils were discernible between samples obtained from healthy non-pregnant women, healthy pregnant women, or cases with GDM (Figure 3A), which was most pronounced 60 min after the HG challenge (Figures S2B,C in Supplementary Material). This increase in NETosis was apparent using either Sytox Green to detect exogenous DNA extruded by isolated neutrophils *in vitro* (Figure 3A, left panel, Figure 3B; Figure S2B in Supplementary Material), fluorescent immunohistochemistry for MPO (Figure 3A, right panel, Figure 3D; Figure S2C in Supplementary Material), as well as by examining for NET products in matching serum samples (Figure S2E in Supplementary Material). As previously observed, this increase in spontaneous NETosis was paralleled by a similar increase in primed pro-NETotic neutrophils, identified by posttranslational histone modifications facilitating chromatin unraveling (citH3^+^) (Figure 3C; Figure S2D in Supplementary Material).

It was also noted that under these conditions, the expression of the NE gene was significantly enhanced in cases with GDM, especially at the 60’ time point of the OGTT assay (Figure 3E).

### Hyperglycemia Promotes NETosis in Coculture with Trophoblast Cells

**Figure 4:**
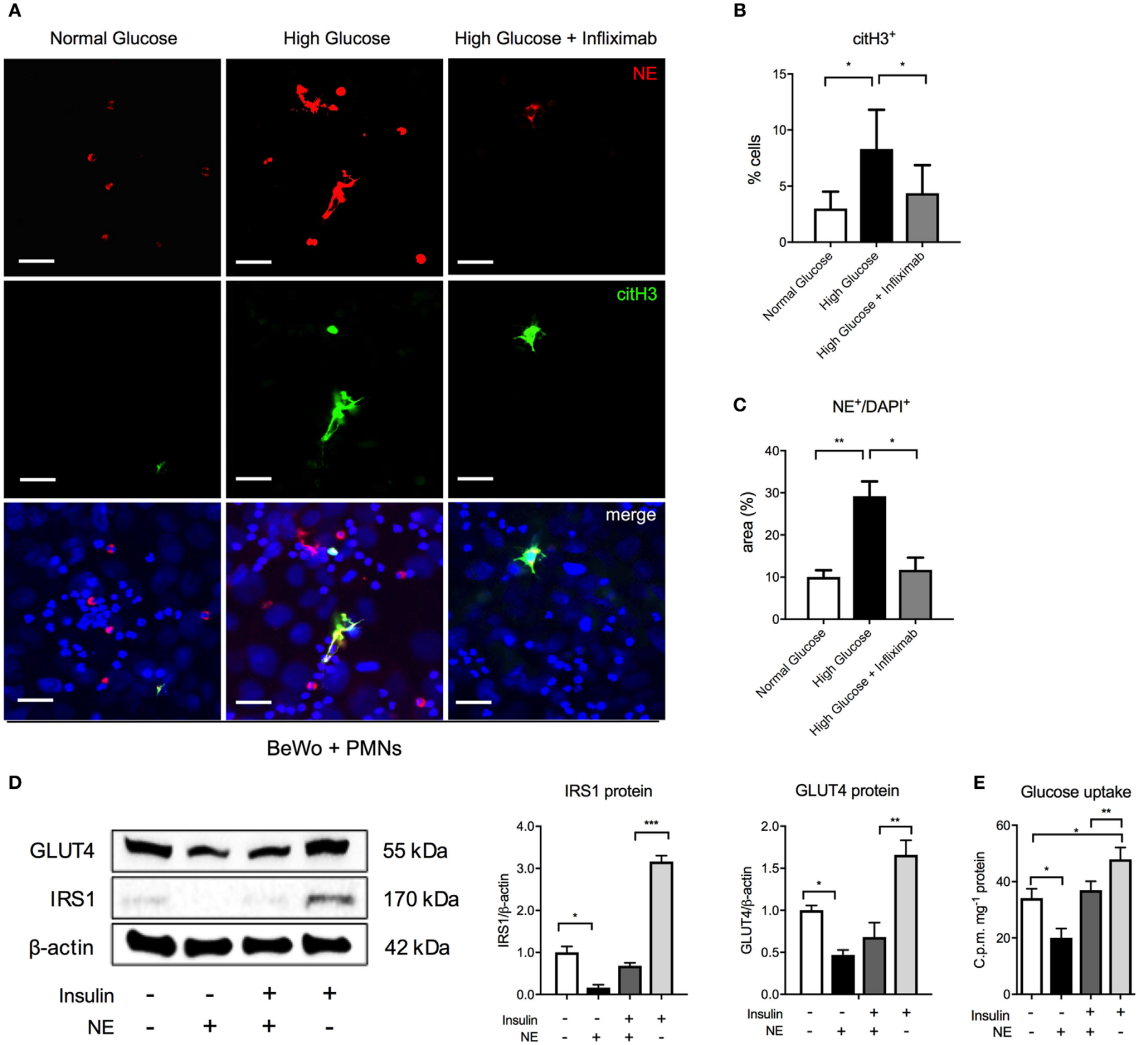
Hyperglycemia triggers neutrophil activation and NETosis in cocultures of isolated control neutrophils and BeWo trophoblast cells. **(A)** Immunofluorescence staining for neutrophil elastase (NE) (red), citH3 (green), and DNA (blue) in polymorphonuclear neutrophil (PMN)–BeWo cell coculture after incubation of PMNs for 3 h with BeWo cells treated with normal glucose (7 mM), high glucose (25 mM), and infliximab (5 µg/ml) for 12 h. Scale bars: 50 µm. **(B,C)** Morphometric analysis of extracellular citH3+ and NE (NE^+^/DAPI^+^) in neutrophils (suggestive of activated and NETing neutrophils) as in **(A)**. **(D)** Western blot and densitometric analysis of IRS1, GLUT4 and β-actin protein expression levels in BeWo cells incubated with NE (80 nM) and/or insulin (100 nM) **(E)** Radiometric Glucose Uptake in BeWo cells incubated as in **(D)**. RFU, relative fluorescence units; AU, arbitrary units. Data are presented as mean ± SEM (*P < 0.05, **P < 0.01, ***P < 0.001) (one-way ANOVA followed by Tukey’s multiple comparison post-test). All experiments were performed at least four times in triplicates with consistent results.

We, next, examined this interplay between hyperglycemia and TNF-α in a coculture experiment, using BeWo trophoblast-like cells and freshly isolated control neutrophils (Figure 4). Here, we observed that HG conditions (25 mM) promoted neutrophil activation, identified by staining for citH3 (Figures 4A,B), as well as enhanced NET formation (Figures 4A,C) identified by co-staining of extruded nuclear material for NE and citH3. Both neutrophil priming and NET formation were reduced by addition of the TNF-α antagonist, infliximab (Figures 4A–C). In this context, it is of interest that infliximab treatment not only reduced the number of NETs but also had a noticeable influence on their size and dimensions (Figure 4A).

### Degranulation of NE Leads to Degradation of IRS1 in Adjacent BeWo Cells

Externally liberated NE has been shown to have a profound effect on neighboring tissues in cancer cells, where its uptake led to the degradation of IRS1, thereby uncoupling the PI3K pathway from the negative regulatory effect of IRS1, thereby leading to uncontrolled growth (36). In a similar manner, exogenous NE was shown to interact with hepatocytes in a murine model for diabetes, where it also enzymatically degraded IRS1 (37), thereby promoting glucose intolerance. For this reason, we examined the influence of exogenous NE on BeWo cells. First, the optimal incubation period of NE with BeWo was determined by monitoring cell viability by means of a MTT assay. Influence of NE on BeWo indicated that akin to the report on cancer cells (36), exogenous NE leads to a significant degradation of IRS1 protein (Figure 4D). This effect of NE is also evident in cultures treated with insulin (Figure 4D). The decrease in IRS1 protein levels was associated with a concomitant decrease in glucose transporter type 4 gene (GLUT4), resulting in diminished glucose uptake (Figure 4E). Once again, this effect was also present in insulin-treated cultures, albeit to a lower level than cultures without insulin (Figure 4E).

### Neutrophil Infiltrates in GDM Placentae Are Associated with a Decrease in IRS1 Expression

**Figure 5:**
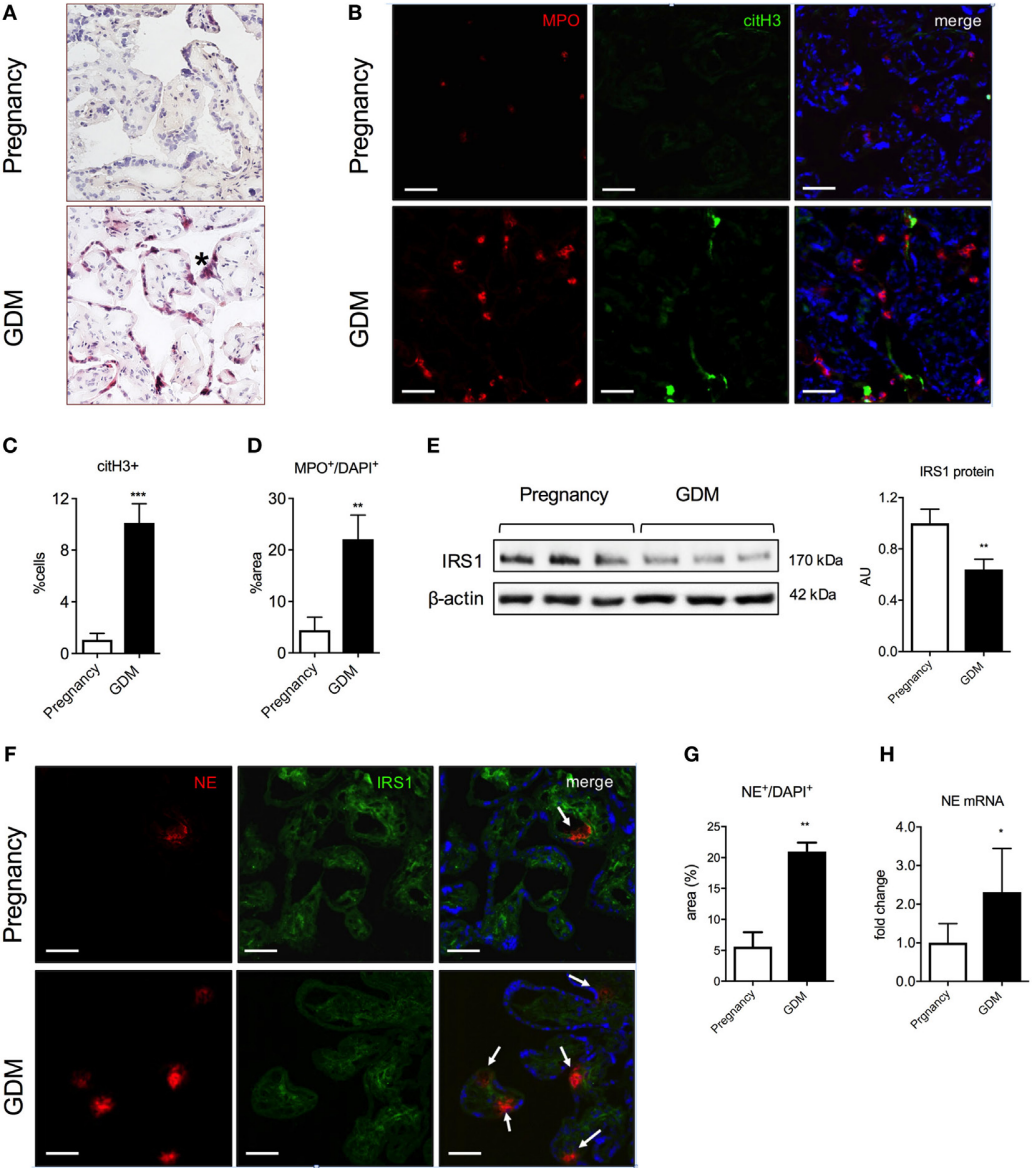
Polymorphonuclear neutrophils lead to excessive neutrophil elastase (NE) release and IRS1 degradation in placenta during gestational diabetes mellitus (GDM). **(A)** H&E staining of placental tissue. (*): syncytial knotting. Scale bars: 50 µm. **(B)** Immunofluorescence staining for MPO (red), citH3 (green), and DNA (blue) of placental tissue collected at the term of pregnancy from controls and GDM women. Scale bars: 50 µm. **(C,D)** Morphometric analysis of extracellular citH3^+^ and MPO^+^ in neutrophils (suggestive of activated and NETing neutrophils) in tissue sections from term placentae of pregnancy and GDM. **(E)** Western blot and densitometric analysis of IRS1 and β-actin protein expression levels in placental tissue as in **(A)**. **(F)** Immunofluorescence staining for NE (red), IRS1 (green), and DNA (blue) of placental tissue as in **(A)**. Scale bars: 100 µm. **(G)** NE protein expression determined by morphometric analysis in placental tissue lysates from pregnancy and GDM. **(H)** mRNA expression level of NE determined by qPCR in placental tissue as in **(G)**. AU, arbitrary unit. Data are presented as mean ± SEM. *P < 0.05, **P < 0.01, ***P < 0.001, ****P < 0.0001 (one-way ANOVA followed by Tukey’s multiple comparison post-test). All experiments were performed at least three times with consistent results.

Our next task was to ascertain whether such an interaction occurs in the placenta of pregnancies affected by GDM. A histological analysis indicated that there were numerous neutrophil infiltrates in the villous tissue in GDM placentae (Figure 5A), which was confirmed by fluorescent immunohistochemistry for MPO and citH3 (Figures 5B–D), suggestive of primed and NETing neutrophils (Figures 5A–D).

We also determined that GDM placentae contained elevated numbers of NE expressing cells at the protein and *de novo* mRNA synthesis (Figures 5F–H). Akin to our observations in BeWo cells, we noted that the elevated NE was associated with a decreased presence of IRS1, as determined both by western blotting (Figure 5E) and fluorescent immunohistochemistry (Figure 5F).

## Discussion

Our data indicate that GDM is associated with an altered neutrophil response, characterized by excessive pro-NETotic activity. In this regard, the enhanced pro-NETotic response in GDM appears somewhat similar to that observed in cases with T1DM or T2DM [recently reviewed in Ref. (15)]. In agreement with previous reports (30, 34), our data also suggest that HG conditions promoted a pro-NETotic state.

A major difference between GDM and the two canonical forms of diabetes is that GDM solely occurs during pregnancy. This factor cannot be ignored, since pregnancy is a unique physiological state known to lead to significant changes in the immune response (39), as is evident from our recent observation that pregnancy itself is associated with a pro-NETotic response (38).

In the context of our current study, it is hence possible that the background contributed by pregnancy may promote a more exaggerated response in cases with GDM. Such an event may contribute to the development of PE, which occurs with increased incidence in cases with GDM, and which is characterized by an overt neutrophil response (9, 10, 12, 20).

Since increased levels of TNF-α have previously been described in GDM placentae or patient sera (49, 51, 52), we examined this aspect in our study. Our data confirm that circulatory TNF-α levels are elevated in our study cohort when compared to the control group. The priming activity of TNF-α on neutrophils is well described (47). To confirm that TNF-α contributes to neutrophil priming in GDM, we used infliximab, a clinically employed antagonist. The addition of this agent to isolated neutrophils treated with GDM plasma significantly diminished the occurrence of NETs in *in vitro* cultures. These findings, hence, suggest that circulatory TNF-α plays a key role in mediating neutrophil activation in cases with GDM.

Furthermore, we observed that TNF-α production was increased in BeWo trophoblast cells exposed to HG (25 mM), thereby supporting previous reports that TNF-α expression is enhanced in GDM placentae (49).

Since TNF-α can promote neutrophil migration (53, 54), this could facilitate increased placental infiltration, where the primed neutrophils would readily undergo NETosis or degranulation.

Although others and we have previously observed increased numbers of NETs in preeclamptic placentae (20, 22), this was not the focus of our current study.

Rather we were intrigued by recent reports indicating that NE released by degranulation can have profound effects on surrounding tissues (36, 37). In the first of these pivotal reports, it was shown that exogenously liberated NE uncoupled tumor cell signaling *via* the degradation of IRS1, leading to deregulated proliferation (36). This was extended upon in a murine diabetes model where it was shown that exogenous NE led to insulin resistance in hepatocytes and contributed to inflammation-induced metabolic disease (37).

In this context, our data closely mirror the observation in tumor cells (36), in that exogenous NE added to BeWo degraded IRS1. We determined that the action of NE also led to a decrease in the abundance of the glucose transporter GLUT4 protein, which was associated with a concomitant decrease in glucose uptake.

Such an action of NE on placental tissues may aid in deciphering characteristic changes or alterations in this organ in GDM, which include increased size, enhanced angiogenesis, and most notably significant villous immaturity (48). The latter is clinically highly relevant as it is associated with poor fetal outcome (48). In addition, fibrin thrombi have been detected attached to the syncytiotrophoblast in placentae affected by GDM (48), a feature also detected in cases with PE (55). It is noteworthy that these placental alterations are frequently evident even in well-managed cases with GDM. It will, thus, be of interest to determine what the contribution of neutrophils is concerning GDM-related placental alterations, specifically villous immaturity and thrombotic events in the intervillous space. It will also be interesting to discern how these are related to the onset of PE.

A noteworthy point of concern in our study is that all the GDM cases studied were clinically considered as being well managed with regard to glucose blood levels. As such, the pro-inflammatory condition we detected is present in a subliminal form, ready to be triggered at a low threshold by a subtle immunological change.

Since overt neutrophil activity (56) and excessive NET formation in the placental intervillous space are evident in PE (20), our new data describing analogous findings in cases with GDM may aid in explaining the increased of risk of these pregnant women to develop PE (8–10). Furthermore, since a large proportion of the circulating neutrophil pool in GDM exhibit an excessive pro-NETotic capacity, ready to form NETs rapidly at a moments notice, this could explain why the pathological transformation to PE occurs so abruptly and is so difficult to detect in advance (9, 10).

In summary, our data indicate that GDM is associated with overt neutrophil activity, resulting in placental infiltration, enhanced NET formation, and NE release. The latter has the potential to profoundly alter placental cell biology *via* the enzymatic degradation of key regulatory signal transduction components. In addition, our findings on overt neutrophil activity may pave the way for more precise screening tools to assist with the detection of GDM than the current challenge with HG levels, which have limited utility and lack international standardization (50, 57, 58, 59).

## Ethics Statement

This study was carried out with the approval of the Ethical committee Nordwest und Zentralschweiz, Basel, Switzerland (EKBB 195/13 or EKNZ PB_2016-00611), with written informed consent from all subjects in accordance with the Declaration of Helsinki.

## Author Contributions

MS, FG, and SG performed all experiments. GS and SB contributed to the sample analyses and performed experiments. IH, OL, and EH provided advice for and contributed to the recruitment of the sample donors and submitted the ethical permission. SG and SH devised and directed the study. SG, SR, PH, and SH wrote the manuscript.

## Conflict of Interest Statement

A patent has been filed under the University and University Hospital Basel and the Kantonspital Aarau, Switzerland. The authors declare that the research was conducted in the absence of any commercial or financial relationships that could be construed as a potential conflict of interest.

## Footnotes

^1^www.graphpad.com.

^2^www.eudox.ch.

## Supplementary Material

Figure S1 **(A)** Spontaneous cell-free nucleosome formation in plasma from control, pregnancy, or gestational diabetes mellitus (GDM) patients in the second and third trimester measured in a 3-h time course.

Figure S2 Analysis of **(A)** blood glucose concentration, **(B)** neutrophil extracellular trap (NET) formation from freshly isolated neutrophils by SytoxGreen staining, **(C)** NET formation from freshly isolated neutrophils as measure of MPO+/DAPI+ area, **(D)** priming of freshly isolated neutrophils as measured with citH3+ cells, and **(E)** serum cell-free nucleosomes in control, pregnancy, or gestational diabetes mellitus (GDM) patients during the oral glucose tolerance test (OGTT); analysis performed before the test (0 min) and 60 and 120 min during the test.
