# Supplementary material for: Gestational Diabetes Mellitus Is Associated with Altered Neutrophil Activity
Source: Front Immunol. 2017 Jun 14;8:702. doi: 10.3389/fimmu.2017.00702 (PMC5469883; doi:10.3389/fimmu.2017.00702)
Supplement: Supplementary file 1 [file Table_1.PDF]

	Non Pregnant Controls	Pregnant donors	Pregnant donors with GDM
**OGTT**	2	4	4
**Total**	10	10 (IIT: 6 IIIT: 4)	9 (IIT: 7 IIIT: 2)
**Age**	33	34	34.5
**Week of gestation**	-	24+5	25+6
**Week of delivery**	-	39+1	38+6
