# Supplementary figures and images for: Gestational Diabetes Mellitus Is Associated with Altered Neutrophil Activity
Source: Front Immunol. 2017 Jun 14;8:702. doi: 10.3389/fimmu.2017.00702 (PMC5469883; doi:10.3389/fimmu.2017.00702)

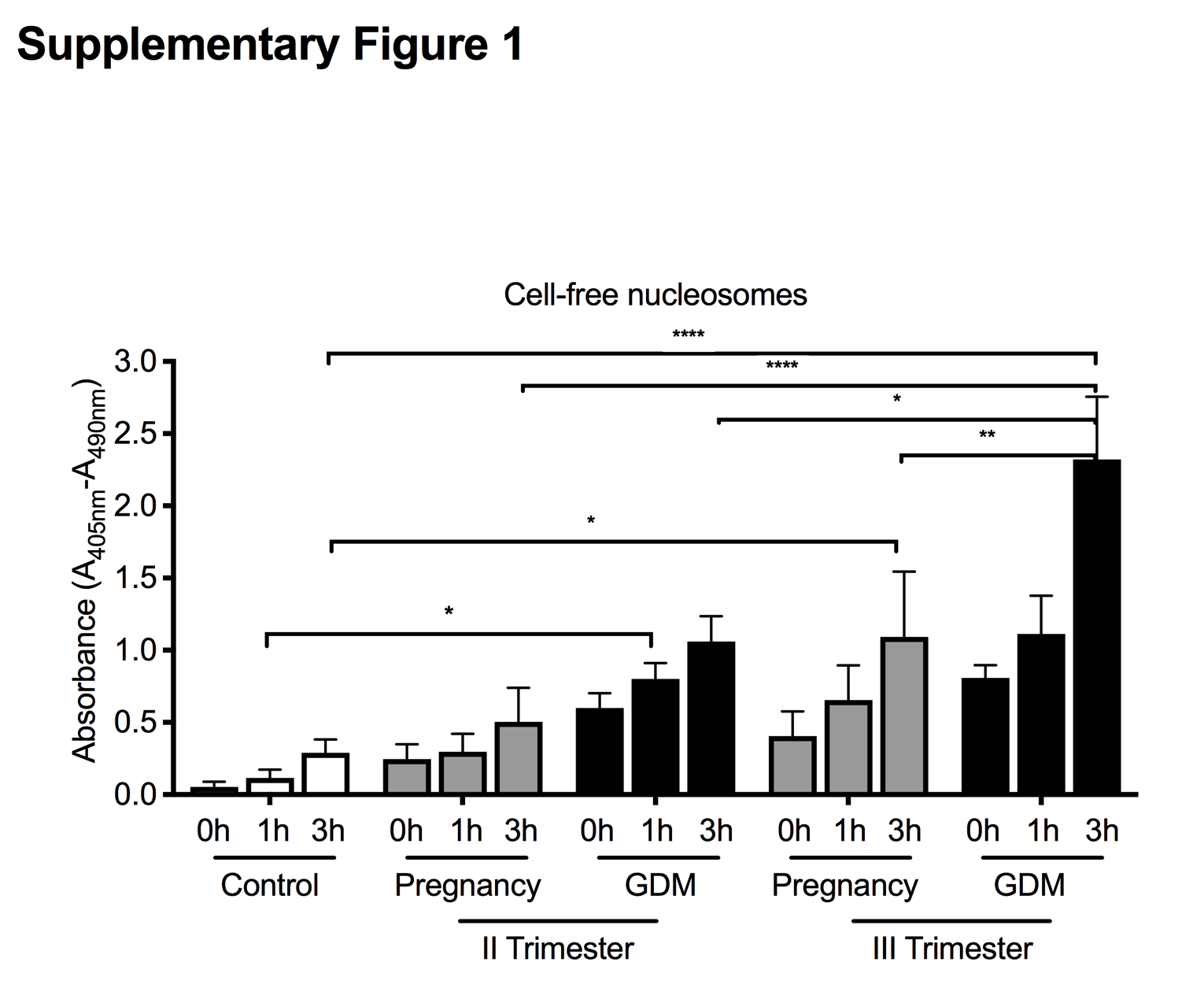

Supplement: Figure S1 — (A) Spontaneous cell-free nucleosome formation in plasma from control, pregnancy, or gestational diabetes mellitus (GDM) patients in the second and third trimester measured in a 3-h time course. [file Image_1.TIFF]

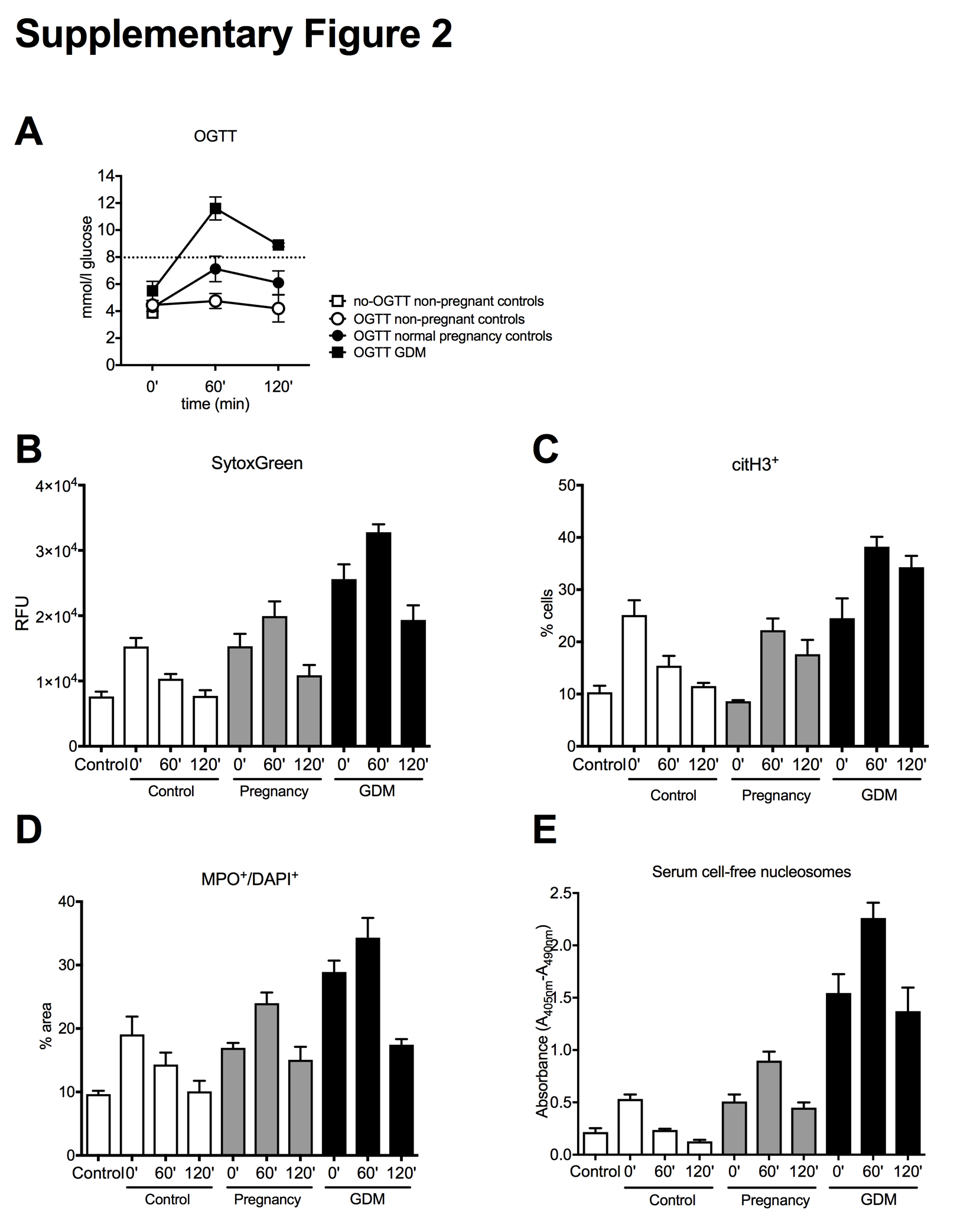

Supplement: Figure S2 — Analysis of (A) blood glucose concentration, (B) neutrophil extracellular trap (NET) formation from freshly isolated neutrophils by SytoxGreen staining, (C) NET formation from freshly isolated neutrophils as measure of MPO+/DAPI+ area, (D) priming of freshly isolated neutrophils as measured with citH3+ cells, and (E) serum cell-free nucleosomes in control, pregnancy, or gestational diabetes mellitus (GDM) patients during the oral glucose tolerance test (OGTT); analysis performed before the test (0 min) and 60 and 120 min during the test. [file Image_2.TIFF]
